# In Vitro and In Vivo Effects of *Gracilaria verrucosa* Extracts on Osteoclast Differentiation

**DOI:** 10.3390/jcm6030032

**Published:** 2017-03-14

**Authors:** Kwang-Jin Kim, Yong-Jin Lee, Yun-Ho Hwang, Kyung-Yun Kang, Sung-Tae Yee, Young-Jin Son

**Affiliations:** Department of Pharmacy, Sunchon National University, Jeonnam, Suncheon 57922, Korea; mastiffk@naver.com (K.-J.K.); yojilee@gmail.com (Y.-J.L.); hyh7733@naver.com (Y.-H.H.); kang8404@nate.com (K.-Y.K.); sungtae@sunchon.ac.kr (S.-T.Y.)

**Keywords:** bone, osteoporosis, osteoclast, receptor activator of nuclear factor-κB ligand (RANKL), nuclear factor of activated T-cells, cytoplasmic 1 (NFATc1), in vivo, seaweed, *Gracilaria verrucosa*

## Abstract

Bone remodeling, a physiological process characterized by bone formation by osteoblasts and bone resorption by osteoclasts, is important for the maintenance of healthy bone in adult humans. Osteoclasts play a critical role in bone erosion and osteoporosis and are bone-specific multinucleated cells generated through differentiation of monocyte/macrophage lineage precursors. Receptor activator of NF-κB ligand (RANKL) has been reported to induce osteoclast differentiation. In this study, we explored whether *Gracilaria verrucosa* extracts (GE) could affect RANKL-mediated osteoclast differentiation. GE significantly inhibited RANKL-activated osteoclast differentiation by inhibiting protein expression of c-fos and nuclear factor of activated T-cells, cytoplasmic 1 (NFATc1), vital factors in RANKL-mediated osteoclastogenesis. In addition, GE attenuated ovariectomy-induced bone loss in mice. In summary, GE can prevent osteoclastogenesis and hormone-related bone loss via blockage of c-fos-NFATc1 signaling. Our results suggest that GE may have therapeutic potential in the treatment of postmenopausal osteoporosis.

## 1. Introduction

Bone is continuously being resorbed by osteoclasts and formed by osteoblasts [1,2]. Balancing the activities of osteoclasts and osteoblasts is important in the bone remodeling process. An impaired balance leads to bone diseases such as osteoporosis, Paget’s disease, rheumatoid arthritis, and periodontal disease due to excessive activity and/or an increased number of osteoclasts [3]. Therefore, controlling the activity and number of osteoclasts is important in patients with bone disease.

Osteoclasts are large multinucleated cells of myeloid lineage origin that can degrade the bone matrix and decalcify via proteolytic degradation and decalcification, respectively [4]. They are tartrate-resistant acid phosphatase-positive multinucleated osteoclast cells (TRAP^+^-MNCs) differentiated from macrophages by macrophage colony-stimulating factor 1 (M-CSF) and receptor activator of nuclear factor-κB ligand (RANKL), which are expressed by osteoblasts [5].

RANKL, an osteoclast differentiation factor, is a type II membrane protein and a member of the tumor necrosis factor (TNF) superfamily [6]. The binding of RANKL to its receptor, RANK, on osteoclast precursors trigger the osteoclast differentiation [7]. The RANKL/RANK system directly regulates osteoclastogenesis and bone remodeling. RANKL/RANK signaling ultimately induces nuclear factor of activated T-cells, cytoplasmic 1 (NFATc1), a key factor in osteoclastogenesis [8]. NFATc1 regulates osteoclast differentiation-related genes, such as TRAP, cathepsin K, and dendritic cell-specific transmembrane protein (DC-STAMP) [9,10,11].

Seaweeds are sources of products that are useful in many fields including food technology, biotechnology, and even medicine [12,13]. *Gracilaria verrucosa* is a species of red algae that is used as a food in Japan and Korea and is known to be effective in osteoporosis. In this study, we investigated the effects of the acid extracts of *Gracilaria verrucosa* (GE) on osteoclastogenesis that play a vital role in osteoporosis.

## 2. Experimental Section

### 2.1. Extract of *Gracilaria verrucosa*

An entire plant of *Gracilaria verrucosa* (Inseong, Jangheung, Korea) was washed under running tap water to remove salt after drying at room temperature. The sample was washed and dried until the salt was completely drained. Three liters of water was added to 200 g of the dried sample and allowed to stand for one day. Then, 15 g of citric acid anhydrous and 150 mL of glacial acetic acid was added to the sample, which was then allowed to stand at room temperature for 4 h. The extract supernatant was filtered using Whatman No. 2 filter paper (Sigma-Aldrich, St. Louis, MO, USA) before being transferred into pre-weighed containers, and the sample was concentrated with a rotary evaporator and freeze-dried to produce crude extracts.

### 2.2. Cell Cultures and Osteoclast Differentiation

This study was carried out in strict accordance with the recommendations contained in the Standard Protocol for Animal Study of Sunchon National University (SCNU, Suncheon, Korea). The protocol was approved by the Sunchon National University Institutional Animal Care and Use Committee (IACUC) with Permit No. SCNU IACUC 2016-07. All efforts were made to minimize suffering.

To obtain bone marrow macrophages (BMMs), bone marrow cells (BMCs) were isolated from the femur and tibia of two mice of 5-week-old male imprinting control region (ICR) strain (Damool Science, Daejeon, Korea) by flushing with α-minimum essential medium (α-MEM; Invitrogen Life Technologies, Carlsbad, CA, USA) supplemented with antibiotics (100 units/mL penicillin and 100 µg/mL streptomycin; Invitrogen, Carlsbad, CA, USA). BMMs were obtained from BMCs cultured on a petri dish in α-MEM supplemented with 10% fetal bovine serum (FBS; Invitrogen Life Technologies, Carlsbad, CA, USA) with 30 ng/mL mouse recombinant macrophage colony-stimulating factor (M-CSF; PEPROTECH, Rocky HilI, NJ, USA) for 3 days. BMMs were seeded and cultured in the presence of 10 ng/mL mouse recombinant RANKL (R&D Systems, Minneapolis, MN, USA) and 30 ng/mL M-CSF for 4 days in the presence or absence of extracts.

### 2.3. Cytotoxicity Assay for Extract of *Gracilaria verrucosa*

BMMs were plated in a 96-well plate in triplicate at a density of 10,000 cells/well, and the cells were treated with 30 ng/mL M-CSF and GE. After culturing for 3 days, cell viability was evaluated with a CCK-8 kit (Dojindo Molecular Technologies, Kumamoto, Japan) according to the manufacturer’s protocol.

### 2.4. Tartrate-Resistant Acid Phosphatase Staining Assay

Cultured cells were fixed with 3.7% formalin for 5 min, permeabilized with 0.1% Triton X-100 for 10 min, and stained with TRAP solution (Sigma-Aldrich, St. Louis, MO, USA) for 10 min. TRAP^+^-multinucleated cells (cells with 3 nuclei or more; 3 ≤ *n*) were deemed as mature osteoclasts.

### 2.5. Western Blot Analysis

Western blotting was performed as described previously [14]. Cells were washed with phosphate-buffer saline (PBS) and lysed in a lysis buffer (50 mM Tris-HCl, 150 mM NaCl, 5 mM EDTA, 1% Triton X-100, 1 mM sodium fluoride, 1 mM sodium vanadate, and 1% deoxycholate) supplemented with 1 mM phenylmethylsulfonyl fluoride (PMSF; Bio Basic, Markham, CA, USA) and 5 μg/mL leupeptin (Sigma-Aldrich, St. Louis, MO, USA). The cell lysates were separated by centrifugation at 15,000 rpm for 12 min. The proteins (20 μg) were subjected to 10% sodium dodecyl sulfate-polyacrylamide gel electrophoresis (SDS-PAGE) and then transferred onto a polyvinylidene difluoride (PVDF) membrane (Amersham Biosciences, Piscataway, NJ, USA). The membrane was blocked with 5% skim milk and then incubated overnight at 4 °C with a primary antibody, as indicated. The membrane was washed and then incubated with horseradish peroxidase (HRP)-conjugated secondary antibody. Antibodies against c-Fos, NFATc1, and actin were purchased from Santa Cruz Biotechnology (Dallas, TX, USA). The protein bands were visualized by using MicroChemi 4.2 (DNR Bio-imaging System, Jerusalem, IL, USA) and Super-Signal West Pico Chemiluminescent Substrate (Pierce Chemical, Rockford, IL, USA).

### 2.6. Real-Time PCR

Real-time PCR was performed as described previously [15]. Primers for real-time PCR were designed (Table 1) by using the Primer3 design program [16]. Quantitative PCR was completed by using a real-time PCR detection system (Bio-Rad, Hercules, CA, USA) and TOPreal qPCR 2× PreMIX (Enzynomics, Daejeon, Korea). All tests were run in triplicate, and data were analyzed by the 2^−ΔΔ*CT*^ method.

### 2.7. Ovariectomized-Induced Bone Erosion

Eighteen mice of five-week-old female ICR strain (Damool Science, Daejeon, Korea) were randomly divided into 3 groups of 6 mice and ovariectomized (OVX; *n* = 12) or sham-operated (*n* = 6) by the dorsal approach under general anesthesia. The OVX + GE group was administered GE (100 mg/kg of body weight) orally (*n* = 6), while the other groups were administered distilled water (DW; vehicle) orally for 6 weeks daily beginning one day after surgery. At the end of the 6-week treatment period, body and uterus weight of animals were determined by using an electronic scale. Blood samples were maintained at room temperature for 1 h, and centrifuged at 5000 rpm for 5 min to obtain serum. Serum was separated immediately and stored at −80 °C. Serum calcium levels were measured by using a diagnostic slide kit and an automatic analyzer (Fuji Dri-Chem, Fujifilm, Tokyo, Japan). TRAP-activity (a marker of osteoclasts number and activity) was measured by using a TRAP enzyme-linked immunoassay (ELISA) kit (USCN Life Science, Houston, TX, USA). Femurs were obtained from mice sacrificed by cervical dislocation and were fixed in 3.5% formaldehyde for one day. Fixed femurs were scanned and analyzed with a Quantum GX microCT imaging system (PerkinElmer, Waltham, MA, USA).

### 2.8. Statistical Analysis

All quantitative data are presented as the mean ± standard deviation (SD) of three replicate experiments. Statistical differences were analyzed by applying a Student’s *t*-test. Probability (*p*) values less than 0.05 were considered significant (*p*-values * <0.05, ** <0.01, and *** <0.001).

## 3. Results

### 3.1. Extract of *Gracilaria verrucosa* Inhibits Osteoclast Differentiation from Macrophages

**Figure 1:**
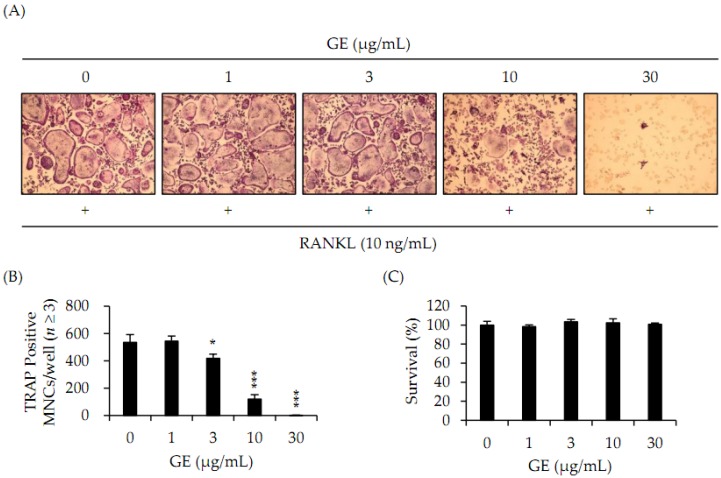
*Gracilaria verrucosa* extracts suppresses osteoclastogenesis. (**A**) Bone marrow macrophages (BMMs) were cultured for 4 days with receptor activator of NF-κB ligand (RANKL) (10 ng/mL) and macrophage colony-stimulating factor (M-CSF) (30 ng/mL) in the presence of the indicated concentrations of GE or vehicle (0.1% distilled water (DW)). Cells were fixed in 3.7% formalin, permeabilized in 0.1% Triton X-100, and stained for TRAP; (**B**) TRAP^+^-multinucleated cells (*n* ≥ 3) were counted as osteoclasts. * *p* < 0.05, *** *p* < 0.001; (**C**) Effect of GE on the viability of BMMs was evaluated by performing CCK-8 assays.

To determine the potential role of GE in osteoclast differentiation, we evaluated the effects of GE on the ability of RANKL to differentiate BMMs. Cells were incubated with a vehicle (0.1% DW) or GE (at indicated concentrations) in the presence of RANKL (10 ng/mL) and M-CSF (30 ng/mL) for 4 days. The formation of TRAP^+^-multinucleated cells (*n* ≥ 3) was induced by RANKL, but GE inhibited such a formation (Figure 1A). GE reduced the number of TRAP^+^-MNCs in a dose-dependent manner, which effect was observed at GE concentrations greater than 3 μg/mL (Figure 1B).

### 3.2. No Cytotoxic Effect of Extract of *Gracilaria verrucosa* on Macrophages

If GE exerts cytotoxicity on the BMMs during culture, osteoclast formation could be inhibited regardless of the effect of GE on osteoclastogenesis. To eliminate this possibility, we performed cell proliferation assays. BMMs were cultured with vehicle (0.1% DW) or GE in the presence of M-CSF (30 ng/mL) for 3 days. As shown in Figure 1C, GE had no cytotoxic effect on BMMs at the concentrations used in this study.

### 3.3. Extract of *Gracilaria verrucosa* Suppresses RANKL-Induced c-Fos and NFATc1 Expression

**Figure 2:**
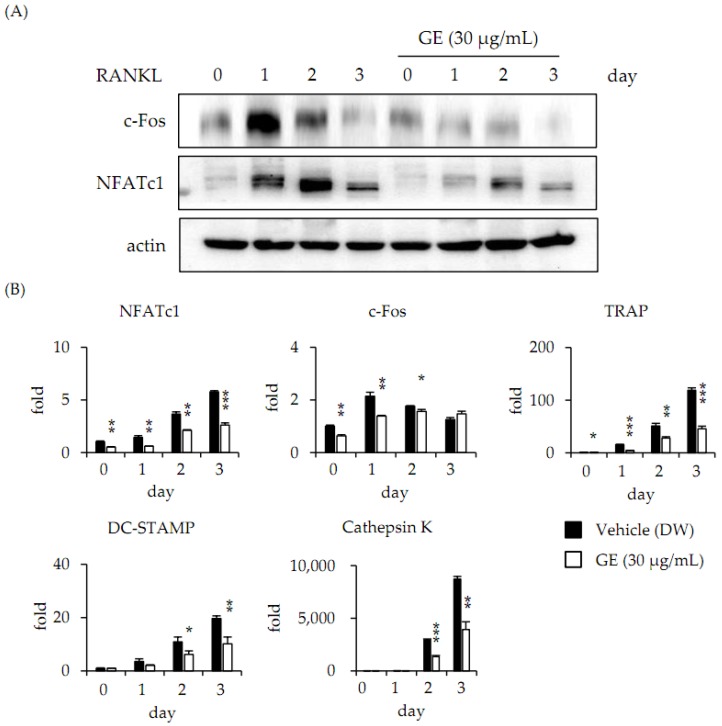
GE inhibits RANKL-induced expression of NFATc1. (**A**) BMMs were pretreated with vehicle (0.1% DW) or GE (30 μg/mL) for 1 h prior to RANKL (10 ng/mL) and M-CSF (30 ng/mL) stimulation at the indicated times. Cell lysates were resolved by sodium dodecyl sulfate-polyacrylamide gel (SDS-PAGE), and Western blotting was performed with anti-c-Fos, anti-NFATc1, and anti-actin antibodies as indicated. (**B**) BMMs were treated with a vehicle (0.1% DW) or GE (30 μg/mL) for 1 h, and RANKL (10 ng/mL) and M-CSF (30 ng/mL) were then treated at the indicated times. Total RNA was then isolated by using Trizol reagent, and mRNA expression levels were evaluated by performing real-time PCR. Glyceraldehyde-3-phosphate dehydrogenase (GAPDH) was used as the internal control. * *p* < 0.05; ** *p* < 0.01; *** *p* < 0.001.

We analyzed the protein expression levels of c-Fos and NFATc1, important regulators of osteoclastogenesis, by Western blotting. RANKL strongly induced protein expression of c-Fos and NFATc1 in osteoclast differentiation, but the c-Fos and NFATc1 protein levels were significantly decreased by GE treatment (Figure 2A). We further confirmed activity of signaling molecules including JNK, p38, ERK, and I-kB, which are known to play a role in the early stage of RANKL-mediated osteoclastogenesis, but GE did not decrease the activation of all of them (Figure S1). Additionally, we observed gene expression of osteoclast-specific markers by undertaking real-time PCR analysis. GE inhibited the mRNA expression of c-Fos and NFATc1 in BMMs treated with RANKL and the mRNA expressions of NFATc1-related genes such as DC-STAMP, cathepsin K, and TRAP in osteoclast differentiation were significantly decreased by GE (Figure 2B).

### 3.4. Effects of Extract of *Gracilaria verrucosa* on Serum Biochemical Markers in Ovariectomized Mice

**Figure 3:**
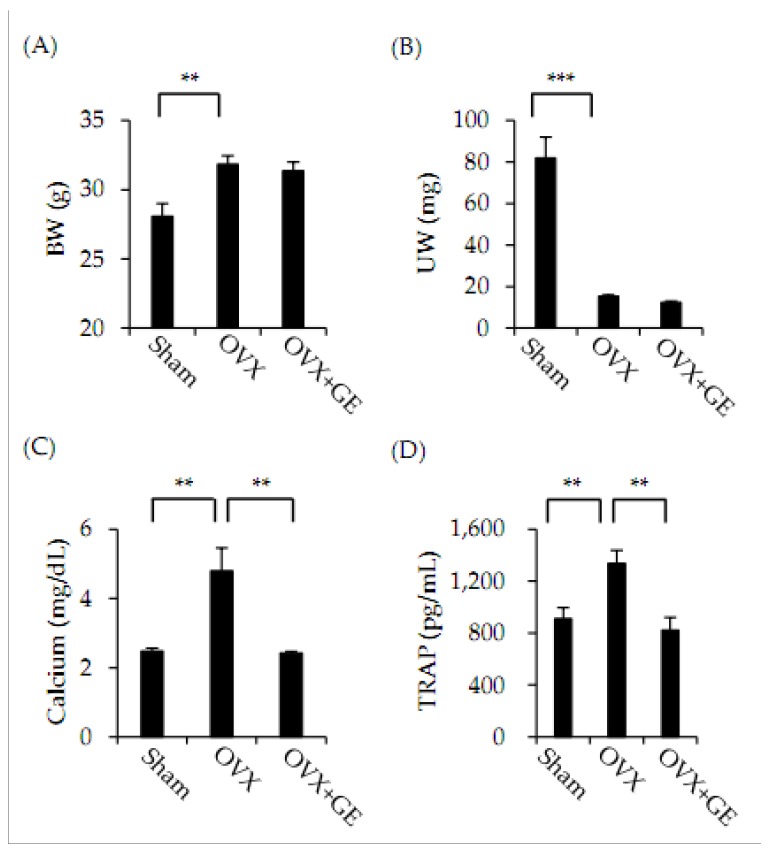
Effect of GE on body weight, uterus weight, and serum biochemical marker levels in ovariectomized (OVX) mice. Mice were treated with vehicle (DW) and GE (100 mg/kg/day) for 6 weeks. (**A**) Body weight (BW) and (**B**) uterus weight (UW). (**C**) Calcium and (**D**) TRAP were analyzed by using ELISA kits as described in materials and methods. Results are presented as means ± standard deviation (SD) (*n* = 6). ** *p* < 0.01; *** *p* < 0.001.

We examined the in vivo effect of GE on bone loss in OVX mice. GE was orally administered daily to OVX mice for six weeks. After six weeks of treatment, we measured body and uterus weights, and assessed serum biochemical marker levels in each mouse. Body weight of the OVX group was significantly greater than the body weight of the sham-operated group, whereas the uterus weight of the OVX group was significantly lower than that of the sham-operated group (Figure 3A,B). In addition, we determined biochemical marker (calcium and TRAP) levels in serum. The serum calcium and TRAP levels were higher in the OVX group than in the sham-operated group (Figure 3C,D).

### 3.5. Extract of *Gracilaria verrucosa* Inhibits Bone Loss in Ovariectomized Mice

**Figure 4:**
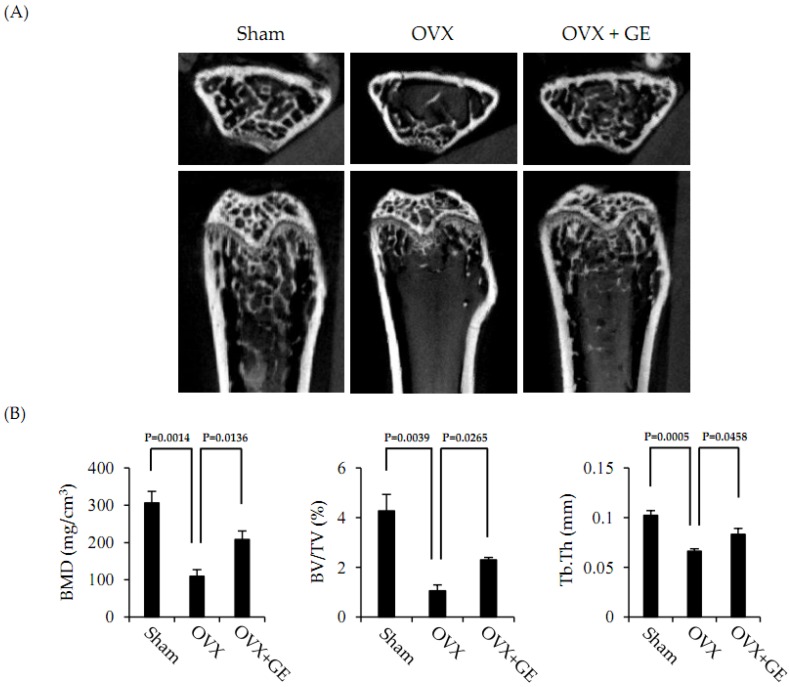
The preventive effect of GE on osteoporosis induced by OVX. (**A**) Representative micro-CT transaxial and coronal images femurs from sham-operated, untreated-OVX, and GE (100 mg/kg)-treated-OVX mice. (**B**) Bone mineral density (BMD; mg/cm^3^), bone volume/tissue volume (BV/TV; %), and trabecular thickness (Tb.Th; mm) were analyzed by using a Quantum GX micro-CT imaging system (PerkinElmer). Results are presented as means ± SE (*n* = 6).

To examine the effects of GE on bone erosion in OVX mice, femurs obtained from mice were analyzed by a micro-computed tomography (CT) system. In trabecular bone in the femur metaphyseal region, bone mass was decreased in OVX mice, and the administration of GE significantly inhibited the trabecular bone loss (Figure 4A). Specifically, ovariectomy-mediated changes in bone mineral density (BMD), bone volume/tissue volume (BV/TV), and trabecular thickness (Tb.Th) were significantly prevented by GE.

## 4. Discussion

Osteoporosis is a major bone disease characterized by low BMD and weakened bone structure. Quality of bone is strongly influenced by the balance of bone-resorbing osteoclast and bone-forming osteoblast activities. Osteoclasts are the only cells that absorb bone and are differentiated from hematopoietic stem cells in the bone marrow [1]. Moreover, they are formed by the fusion of the mononuclear macrophage lineage [4]. RANKL and M-CSF are most affected by osteoclast formation [17].

In this study, we investigated the effect of GE on RANKL-mediated osteoclast differentiation. BMMs were cultured with RANKL and M-CSF in the presence of GE. The GE completely inhibited TRAP^+^-osteoclasts and exhibited no cytotoxicity at concentrations over 30 μg/mL, and this concentration was used in the following experiment. According to this results, GE has a negative effect on osteoclastogenesis with no cytotoxicity to BMMs.

The TNF-family molecule RANKL is a key regulator of osteoclastogenesis. The binding of RANKL to RANK activates signaling molecules such as MAP kinases (MAPKs), c-Fos, and phospholipase Cγ (PLCγ) that ultimately induce the activation of NFATc1 [18,19]. Therefore, in order to understand the molecular mechanism of GE inhibition of osteoclast differentiation, the effect of GE on the protein expression level of c-Fos and NFATc1 was analyzed by Western blotting. Protein expression of c-Fos and NFATc1 was strongly inhibited by GE. However, MAPKs, upper signals of c-Fos, were not affected by GE and did not reduce the degradation of I-kB, which indirectly confirmed the activity of NF-kB [20]. In addition, NFATc1 is an essential transcription factor that regulates osteoclast-specific marker genes such as DC-STAMP, essential for cell-cell fusion, cathepsin K, matrix-degrading enzymes, and TRAP exhibiting bone resorptive activity [21,22]. In this study, we found that GE inhibits RANKL-induced osteoclast differentiation through the downregulation of c-Fos-NFATc1 signaling.

Furthermore, we identified effects of GE in vivo in an OVX mouse model that has been reported to be useful in skeletal studies [23]. GE was orally administered to OVX mice for six weeks. The success of OVX surgery was confirmed by observing significant increases in body weight and marked decreases in uterus weight [24]. In addition, we checked for changes in biochemical markers in serum. Oral administration of GE completely suppressed changes in calcium and TRAP levels. These results indicate that GE inhibits osteoclast differentiation and activation. In addition, we examined the femurs of mice via micro-CT. As shown in Figure 4A, micro-CT revealed that GE treatment had inhibited OVX-induced bone loss. Histomorphometric analysis of femurs confirmed the inhibitory effects of GE on OVX-induced bone loss in vivo (Figure 4B) with GE significantly attenuating the OVX-induced changes in BMD, BV/TV, and Tb.Th, but it was not fully recovered. We anticipate that, if we increase the amount of extract in treatment, the difference in results between the sham group and the GE-treated group will decrease.

In summary, GE inhibits osteoclast differentiation by reducing the expression of NFATc1 through the inhibition of c-Fos expression. Moreover, GE can significantly reduce OVX-induced bone loss in vivo. Therefore, GE could be useful when developing a new osteoporosis drug.

## Figures and Tables

**Table 1:** Primer sequences used in this study.

Gene of Interest	Primer Sequence (5′→3′)	
	Sense	Anti-Sense
NFATc1	GGGTCAGTGTGACCGAAGAT	GGAAGTCAGAAGTGGGTGGA
c-Fos	CCAGTCAAGAGCATCAGCAA	AAGTAGTGCAGCCCGGAGTA
cathepsin K	GGCCAACTCAAGAAGAAAAC	GTGCTTGCTTCCCTTCTGG
DC-STAMP	CCAAGGAGTCGTCCATGATT	GGCTGCTTTGATCGTTTCTC
TRAP	GATGACTTTGCCAGTCAGCA	ACATAGCCCACACCGTTCTC
GAPDH	AACTTTGGCATTGTGGAAGG	ACACATTGGGGGTAGGAACA

Note: NFATc1: nuclear factor of activated T-cells, cytoplasmic 1; DC-STAMP; dendritic cell-specific transmembrane; GAPDH: glyceraldehyde-3-phosphate dehydrogenase.

## Supplementary material

Supplementary File 1
