# Supplementary material for: In Vitro and In Vivo Effects of Gracilaria verrucosa Extracts on Osteoclast Differentiation
Source: J Clin Med. 2017 Mar 14;6(3):32. doi: 10.3390/jcm6030032 (PMC5373001; doi:10.3390/jcm6030032)
Supplement: Supplementary File 1 [file jcm-06-00032-s001.doc]

**Supplementary Information**

**Materials and Methods**

*Western Blot Analysis*

Western blot analysis was performed as described in “Material and Methods.” Antibodies against p-P38, P38, p-ERK, ERK, p-JNK, and JNK were purchased from Cell Signaling technology (Danvers, MA, USA). Antibodies against I-kB were purchased from Santa Cruz Biotechnology (Dallas, TX, USA).


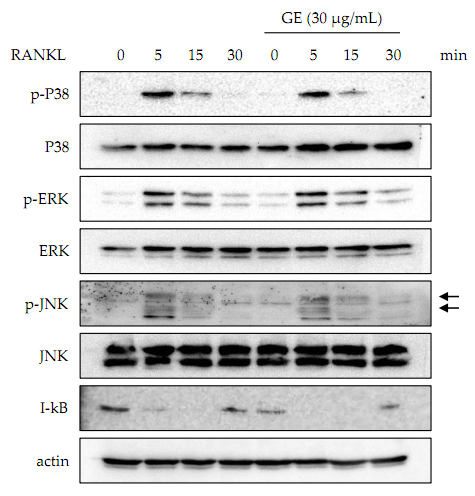


**Figure S1.** Effect of GE on RANKL-induced activation or expression of osteoclast-specific cell signaling molecules. The effects of GE on RANKL-induced phosphorylation of MAP kinases and I-kB were evaluated by Western blot analysis. BMMs were pre-treated with GE (30 μg/mL) 1 h before treatment with RANKL (10 ng/mL).
